# A Randomized Controlled Trial of Conbercept Pretreatment before Vitrectomy in Proliferative Diabetic Retinopathy

**DOI:** 10.1155/2016/2473234

**Published:** 2016-03-13

**Authors:** Xiaochun Yang, Jianbiao Xu, Ruili Wang, Yan Mei, Huo Lei, Jun Liu, Ting Zhang, Haiyan Zhao

**Affiliations:** ^1^Department of Ophthalmology, The First People's Hospital of Yunnan Province, Ocular Fundus Disease Research Center of Yunnan Province, The Affiliated Hospital of Kunming University of Science and Technology, Yunnan 650032, China; ^2^Department of General Surgery, The First People's Hospital of Yunnan Province, Yunnan 650032, China; ^3^State Key Laboratory of Ophthalmology, Zhongshan Ophthalmic Center, Sun Yat-Sen University, Guangdong 510062, China

## Abstract

*Purpose.* To determine the efficacy and safety of preoperative intravitreal conbercept (IVC) injection before vitrectomy for proliferative diabetic retinopathy (PDR).* Methods.* 107 eyes of 88 patients that underwent pars plana vitrectomy (PPV) for active PDR were enrolled. All patients were assigned randomly to either preoperative IVC group or control group. Follow-up examinations were performed for three months after surgery. The primary bioactivity measures were severity of intraoperative bleeding, incidence of early and late recurrent VH, vitreous clear-up time, and best-corrected visual acuity (BCVA) levels. The secondary safety measures included intraocular pressure, endophthalmitis, rubeosis, tractional retinal detachment, and systemic adverse events.* Results.* The incidence and severity of intraoperative bleeding were significantly lower in IVC group than in the control group. The average vitreous clear-up time of early recurrent VH was significantly shorter in IVC group compared with that in control group. There was no significant difference in vitreous clear-up time of late recurrent VH between the two groups. Patients that received pretreatment of conbercept had much better BCVA at 3 days, 1 week, and 1 month after surgery than control group. Moreover, both patients with improved BCVA were greater in IVC group than in control group at each follow-up.* Conclusions.* Conbercept pretreatment could be an effective adjunct to vitrectomy in accelerating postoperative vitreous clear-up and acquiring stable visual acuity restoration for PDR.

## 1. Introduction 

Proliferative diabetic retinopathy (PDR) is a severe and common complication of diabetes mellitus (DM) linked to permanent vision loss or blindness all over the world. Pars plana vitrectomy (PPV) is the cornerstone of management for advanced complications of PDR. However, intraoperative or postvitrectomy vitreous hemorrhage (VH) often occurred to make the visibility too muddy to accomplish surgery favorably. What is more, it could result in retinal detachment or other adverse complications [1, 2].

Angiogenesis is the pathophysiology base of PDR [3]. Vascular endothelial growth factor (VEGF) has been confirmed to be a key driver of the neovascularization, vascular permeability, diabetic macular edema, and PDR [4–6]. The present success of therapies that focus on VEGF antagonist demonstrates that it is a safe and effective adjunct for the management of recurrent VH during or after vitrectomy in PDR [7–9].

The VEGF family consists of VEGF-A, VEGF-B, VEGF-C, and VEGF-D and placental growth factor (PIGF), which are related to receptors, VEGF receptor 1 (VEGFR-1), VEGFR-2, and VEGFR-3. VEGF-A can activate both VEGFR-1 and VEGFR-2. Meanwhile, VEGF-B and PIGF only bind to VEGFR-1. Also, VEGF-C and VEGF-D only bind to VEGFR-3 [10]. There are four currently available VEGF antagonists, pegaptanib, ranibizumab, bevacizumab, and aflibercept, which have been developed and tested in patients with PDR. However, the first three agents had been found to bind VEGF-A only and lasted for only a short time. Aflibercept, binding to more members (VEGF-A, VEGF-B, and PIGF), has shown good effectiveness in retinal neovascularization [11]. Conbercept, a new medicine of KH902, is a humanized, soluble, VEGFR protein which comprises extracellular domain 2 of VEGFR-1 and extracellular domains 3 and 4 of VEGFR-2, all of which are combined with the Fc region of human immunoglobulin G1 simultaneously. Previous studies have demonstrated that extracellular domain 4 of VEGFR-2 can enhance the three-dimensional structure and efficiently advance dimerization [12]. Also, preclinical studies have presented higher affinity of KH902 for VEGF than bevacizumab [13]. Moreover, recent phase 1 studies have shown an antiangiogenic effect of KH902 for choroidal neovascularization caused by age-related macular degeneration [14]. However, the effect of conbercept on diabetic vitrectomy has not been previously reported. In this study, we sought to compare the therapeutic efficacy of vitrectomy combined with conbercept pretreatment vitreous vitrectomy for DM patients with active PDR.

## 2. Patients and Methods

### 2.1. Study Design

The study was a prospective, single-center, single-dose, randomized controlled clinical trial conducted on subjects with active PDR at the First People's Hospital of Yunnan Province, Kunming, Yunnan, China, from March 1, 2014, to January 7, 2015. Protocol was reviewed and approved by the First People's Hospital of Yunnan Province ethics committee. Patients provided written informed consent before study entry.

### 2.2. Patients

The study population consisted of male or female patients aged ≥18 years with type 1 or 2 DM (as per the American Diabetes Association or World Health Organization guidelines), hemoglobin (Hb) A1c ≤ 10%, and visual loss only due to nonabsorbent VH, fibrovascular proliferation with vitreoretinal adhesions or tractional retinal detachment (TRD) dependent on clinical ocular findings and accessory examinations who had to undergo vitrectomy. The control included a range of severities of PDR similar to the criteria set for the study group but received PPV only during the same period. The contralateral eye fulfilling the enrollment criteria and requiring PPV was taken into analysis too if the study eye accomplished all the follow-up.

Key exclusion criteria were history of coagulopathy, hypertension, and stroke or transient ischemic attack; intraocular treatment with corticosteroids, anti-VEGF, or intraocular surgery at any time; confirmed intraocular pressure (IOP) ≥21 mmHg, glaucoma, or iris neovascularization in either eye; severe retinopathy with long-acting gas or silicone oil usage during surgery; a follow-up period of less than 3 months.

### 2.3. Intervention

Patients were randomized into two categories. In preoperative intravitreal conbercept (IVC) group, patients received intravitreal conbercept (0.5 mg/0.05 mL; Chengdu Kanghong Biotech, Inc., Chengdu, Sichuan, China) injection 3 days before PPV. Conbercept was drawn into a 1 mL syringe and injected into the vitreous cavity through a 30-gauge needle following a standard protocol [15]. All patients underwent 23-gauge PPV as described previously [16], including fibrovascular membranes and vitreous dissection, endolaser photocoagulation, peripheral retinal cryotherapy, and endodiathermy as required. Intraoperative hemostasis was obtained by increasing the infusion bottle height or endodiathermy. Any initial bleeding site was checked carefully until no fresh bleeding could be detected. Patients in control group received PPV directly. Two experienced retina surgeons (Xiaochun Yang and Huo Lei) performed all injections and surgeries. The surgeons were masked to randomization when performing the injection and surgery.

### 2.4. Study Assessments and Activities

Subjects were evaluated at each follow-up visit using the following assessments: intraocular pressure (IOP), best-corrected visual acuity (BCVA), slit-lamp examination, ophthalmoscopy, fundus photographs, and B-Scan ultrasonography. BCVA was tested by Snellen equivalent. All patients underwent examinations at baseline and during the first three days after surgery, then biweekly for one month, and then monthly for three months. Clinical managements were given if postoperative complications occurred. The observers were masked for investigating and grading.

The primary bioactivity measures were severity of intraoperative bleeding, incidence of recurrent VH, vitreous clear-up time, BCVA levels, and improvement of BCVA. The secondary safety outcome measures included intraocular pressure (IOP), endophthalmitis, rubeosis, tractional retinal detachment, and systemic adverse events. Extent of vitreoretinal adhesion was defined as the previous classification system [17] and graded from postoperative day 1 until the final follow-up. Absence of any adhesion was grade 0; focal adhesion less than of 3 sites was grade 1; broad adhesion of ≥1 site or adhesion at the disc, macular, or vascular arcade was grade 2; and vitreoretinal adhesion extending to the periphery was grade 3. Severity of intraoperative bleeding was defined as follows: grade 0 (no bleeding), grade 1 (minor bleeding that stopped either spontaneously or by transient bottle elevation), grade 2 (moderate bleeding resulting in broad sheaths of clots requiring endodiathermy to stop bleeding), and grade 3 (thick clot formation covering half or more of the posterior pole or interfering with the surgical plane) [18]. Any new episode of grade 1 or more VH occurring more than 1 week after surgery was considered as recurrent VH, which was taken as early recurrent VH if incidence ≤ 4 weeks, otherwise as late recurrent VH. The severity of VH was assessed by the VH grading system [9]. No VH was grade 0; mild VH with visible fundus details, but with difficulty evaluating the retina nerve fiber layer or small vessels, was grade 1; moderate VH with visible optic disc and large vessels was grade 2; severe VH with faint fundus reflex, only optic disc visible, was grade 3; very severe VH with no fundus reflex and no view of the fundus was grade 4. Vitreous clear-up time was defined as the interval between the end of surgery and the time at which the vitreous cleared up completely. Three levels of visual acuity (VA) were classified: low (≤one-meter counting fingers), moderate (>one-meter counting fingers, but <20/200), and good (≥20/200) [19].

### 2.5. Statistical Methods

Baseline characteristics of all the patients were collected and were analyzed using an intent-to-treat analysis with IBM SPSS Statistics 19.0 software (IBM SPSS Inc., Chicago, USA). Data were presented as mean ± SD. Continuous variables were analyzed using variance or Kruskal-Wallis test, and statistical analysis of the data was performed using Chi-squared test or Fisher exact test. A threshold of *P* value less than 0.05 was set for statistical significance.

## 3. Results

### 3.1. Baseline Characteristics

**Figure 1:**
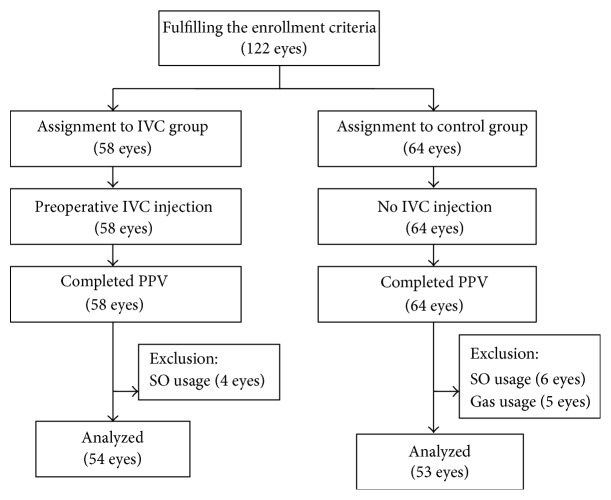
Chart showing participants flow. IVC: intravitreal conbercept; PPV: pars plana vitrectomy; SO: silicone oil.

A total of 122 eyes of 96 patients were enrolled in the study and assigned randomly into two groups: 58 eyes in IVC group and 64 eyes in control group. However, 4 eyes in IVC group were excluded for silicone oil usage and 11 eyes in control group were excluded: 6 cases for silicone oil usage and 5 cases for gas usage. Finally, 54 eyes of 46 patients were included in preoperative intravitreal conbercept group, and control group consisted of 53 eyes of 42 patients (Figure 1). The baseline demographic data is summarized in Table 1. There were no statistically significant differences in sex, age, type of diabetes, HbA1c, duration of diabetes, systemic hypertension, study eye, previous history of laser, lens status, pathogeny, and extent of vitreoretinal adhesion grade between the two groups. All the patients had attached retina in the follow-up period of three months.

### 3.2. Surgical Procedures and Outcomes

**Figure 2:**
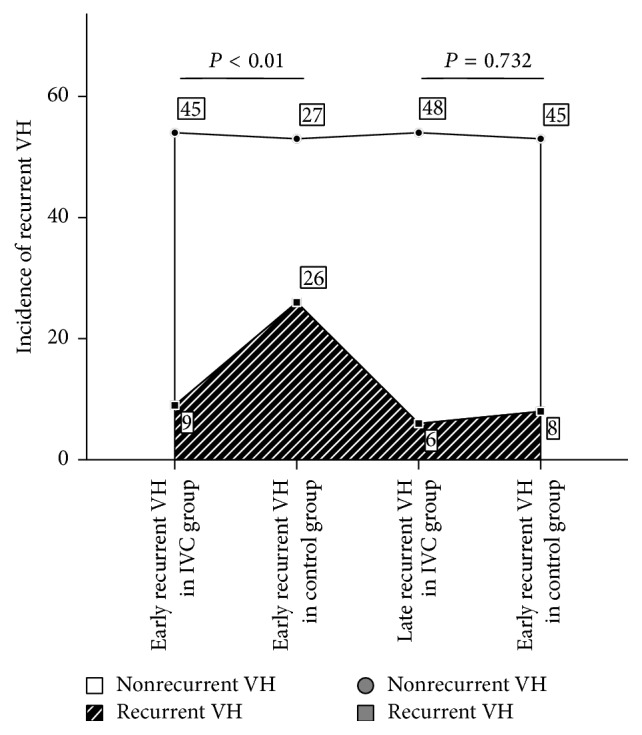
Bar graph showing incidence of early and late recurrent VH with or without conbercept pretreatment after surgery. Subgroup pairwise analysis showed significant differences in early recurrent VH incidence between preoperative IVC group and control group. However, there was no statistically significant difference in late recurrent VH in both groups (*P* < 0.001, *P* = 0.732, resp.). VH: vitreous hemorrhage; IVC: intravitreal conbercept.

The surgical procedures and outcomes are shown in Table 2 and Figure 2. During surgery, grade 3 intraoperative bleeding was added up to 3 of 54 eyes (5.56%), grade 2 up to 10 of 54 eyes (18.51%), grade 1 up to 27 of 54 eyes (50.00%), and grade 0 up to 14 of 54 eyes (25.93%) in preoperative IVC group. Comparing to this, grade 3 intraoperative bleeding was added up to 10 of 53 eyes (18.87%), grade 2 up to 22 of 53 eyes (41.51%), grade 1 up to 14 of 53 eyes (26.42%), and grade 0 up to 7 of 53 eyes (13.21%) in control group. The difference in the severity of intraoperative bleeding was statistically significant (*P* = 0.002).

Nine of fifty-four eyes (16.67%) in preoperative IVC group exhibited early recurrent VH after surgery, two with the severity of grade 2 and seven of grade 1. In control group, the early recurrent VH incidence was 49.05% (26 of 53 eyes). Severity of those twelve eyes was one of grade 4, two of grade 3, eight of grade 2, and fifteen of grade 1. Pairwise subgroup analysis comparing both groups showed significant decrease in early recurrent VH rate and severity in preoperative IVC group compared with control group (*P* < 0.001, resp.). The late recurrent VH after surgery was 6 of 54 eyes (11.11%) in preoperative IVC group. Severity of recurrent VH in the six eyes was two of grade 2 and four of grade 1. In control group, 8 of 53 eyes (15.01%) had late recurrent VH, among which one was grade 3, three were grade 2, and four were grade 1. There were no statistical differences between the two groups in late recurrent VH rate and severity (*P* = 0.973, *P* = 0.732, resp.).

**Figure 3:**
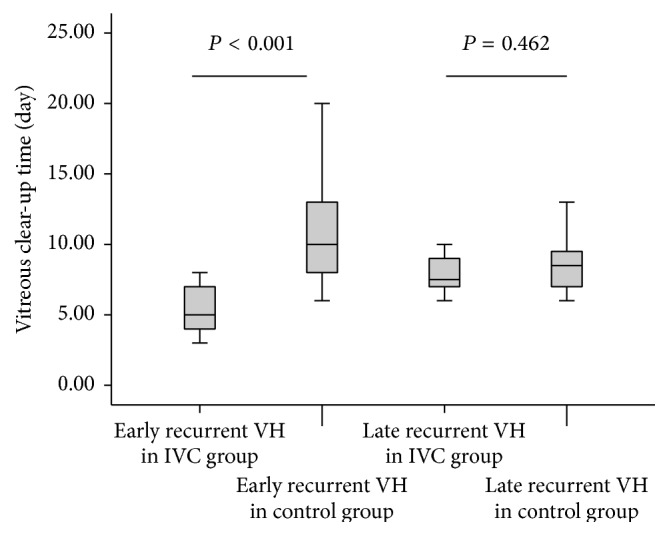
Graph showing vitreous clear-up time of early and late recurrent vitreous hemorrhage in participants with or without conbercept pretreatment. Subgroup pairwise analysis showed significant differences between IVC group and control group in early recurrent VH clear-up time (*P* < 0.001), but no statistical differences in late recurrent VH clear-up time between the two groups (*P* > 0.05). VH: vitreous hemorrhage; IVC: intravitreal conbercept.

The average vitreous clear-up time of early recurrent VH was significantly shorter in preoperative IVC group (5.44 ± 1.94 days) than that in control group (11.31 ± 4.24 days; *P* < 0.001). There was no significant difference in vitreous clear-up time of late recurrent VH between the two groups (7.83 ± 1.47 days, 8.62 ± 2.20 days, resp.; *P* = 0.462; Figure 3).

**Figure 4:**
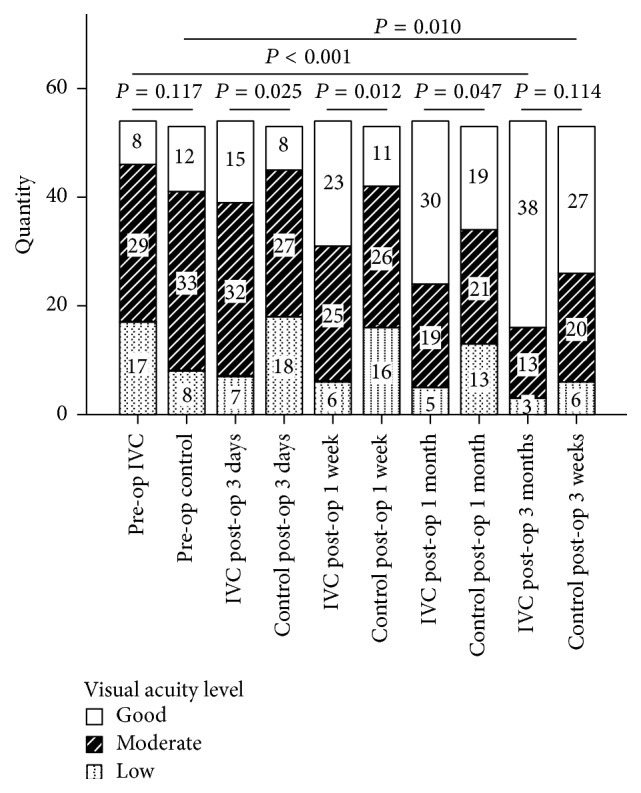
Bar graph showing best-corrected visual acuity levels in participants with or without conbercept pretreatment preoperatively and at 3 days, 1 week, 1 month, and 3 months after surgery. Subgroup pairwise analysis showed significant differences of visual acuity levels at 3 days (*P* = 0.025), 1 week (*P* = 0.012), and 1 month (*P* = 0.047) after surgery between preoperative IVC group and control group. However, there was no statistically significant difference at 3 months (*P* = 0.114) after surgery between the two groups. Compared with the preoperative period, visual acuity levels had a significant increase at 3 months after surgery in both groups (*P* < 0.001, *P* = 0.010, resp.). IVC: intravitreal conbercept; pre-op: preoperative; post-op: postoperative.

In the analysis of BCVA levels at each follow-up period, participants that received pretreatment of conbercept had much better BCVA at 3 days, 1 week, and 1 month after surgery than those in control group (*P* < 0.05, resp.). However, there was no statistically significant difference at 3 months after surgery between the two groups (*P* = 0.114). Moderate VA accounted for the most at 3 days (32/54 eyes, 59.26%) and 1 week (25/54 eyes, 46.30%) after surgery and good VA accounted for the most at 1 month (30/54 eyes, 55.56%) and 3 months (38/54 eyes, 70.37%) after surgery in preoperative IVC group. In control group, moderate VA accounted for the most at 3 days (27/53 eyes, 50.94%), 1 week (26/53 eyes, 49.05%), and 1 month (21/53 eyes, 39.62%) after surgery and good VA accounted for the most at 3 months (27/53 eyes, 50.94%) after surgery (Figure 4). Percentage of VA at good level and BCVA improvement in IVC group tended to be larger than those in control group during all the follow-up periods (*P* < 0.05, resp.). The percentage of VA at good level in IVC group was 14.81% (8/54), 27.78% (15/54), 43.59% (23/54), 55.56% (30/54), and 70.37% (38/54) at baseline, 3 days, 1 week, 1 month, and 3 months after surgery, respectively. In control group, the percentage was 22.64% (12/53), 15.09% (8/53), 20.75% (11/53), 35.85% (19/53), and 50.94% (27/53), respectively, at each follow-up period. Compared with baseline, final BCVA at 3 months after surgery had a significant increase in both groups. Ratio of BCVA improvement at 3 months after surgery was 90.74% (14/54) and 73.58% (39/53) in IVC group and control group, respectively (*P* = 0.20).

### 3.3. Adverse Events

Transient increase of IOP was observed in IVC group which was related to the injection procedure but not to the surgery. The average IOP immediately before conbercept injection and after injection was 16.74 ± 3.21 mmHg and 23.55 ± 4.62 mmHg, respectively. All the IOP lasted for 1-2 hours after injection and then normalized without any special treatments. No endophthalmitis, rubeosis, and TRD progression were observed during the follow-up period in all the cases. No systemic serious adverse events (cardio- and cerebral vascular accident) were observed.

## 4. Discussion

Postoperative recurrent VH strongly hinders visual rehabilitation. VEGF has been suggested to be an important stimulus for neovascularization. Previous reports have suggested that intravitreal injection of KH902 had marked inhibitory effects on angiogenesis both in vivo and in vitro [20]. This prospective randomized study was conducted to investigate the incidence and clearance speed of recurrent VH after PPV in patients with active PDR when administered with a single intravitreal injection of conbercept at the dose recommended of 0.5 mg before surgery.

The results from the present study demonstrated that adjunctive delivery of IVC significantly reduced incidence and severity of intraoperative VH in diabetic vitrectomy. The reason might be related to rapid regression of retinal neovascularization so as to dissect fibrovascular membranes more easily.

It was shown to be lower and lighter in IVC group as to analysis of incidence of early postoperative VH in the present study where only 16.67% patients had mild to moderate VH. Comparing to this, 49.05% of the patients had mild to very severe VH in controls. It could be suggested that IVC may facilitate reducing and alleviating the incidence of early postoperative hemorrhage in diabetic vitrectomy. However, comparison of the two groups showed that adjunctive use of IVC did not significantly reduce late postoperative VH incidence.

It is usually difficult to determine the source of early and late postoperative VH after vitrectomy for PDR. Some reports showed that the early postoperative VH was relevant to dissection of fibrovascular membranes in surgery which occurred typically within one week of surgery [21]. Also, recurrent bleeding from the initial bleeding site, released blood cell from the retained peripheral vitreous base, bleeding from surgically injured retinal tissue, and increased inflammation which has the undesirable effect of increasing the vascular permeability [22] may be the other factors. It has been suggested that VEGF plays an important role in arousing retinal angiotelectasis, basement membrane thickness, and Evans blue dye permeability in diabetes [23]. Hence, conbercept, a strong anti-VEGF agent, pretreatment surely facilitates reducing postoperative bleeding early after surgery due to the regression of neovascularization, cessation of hemorrhage from all potential bleeding sources, and reintegration of retinal vascular tissue. However, it is unlikely to prevent early postoperative bleeding from retained peripheral vitreous base and injured vessels mechanically during surgery. Also, effects of anti-VEGF agents injection are known to be short-term effects with reperfusion of abnormal vessels in time [24]. With vitrectomy, almost all the amount of conbercept injected preoperatively should have been removed during vitrectomy; thus, postoperative effect by remnant conbercept might be negligible. Hence, the anti-VEGF effect of IVC injected before surgery would have lasted for a shorter time, resulting in late VH incidence. This is comparable to the study by Ahn et al., where intravitreal bevacizumab (IVB) injection was given to patients with PDR before 23-gauge PPV [9]. According to that study, the incidence of VH was 22.2% in IVB group and 32.4% in control group at 1 month after surgery. The incidence of early recurrent VH in IVC group in the current study was significantly lower than in IVB group in that report (16.67% versus 22.2%), especially comparing to the control group (49.05% versus 32.4%). The difference of anti-VEGF strength between conbercept and bevacizumab may explain the result. However, other reasons, including different baseline of systemic and ocular profile, different surgical techniques, and small sample size, should be taken into account.

Different from early postoperative VH, late recurrent VH in that report by Ahn et al. [9] was 11.1% in IVB group, which seemed to be similar to the current study (11.11% in preoperative IVC group) at the same time point after surgery. Meanwhile, there was no statistical difference between IVC group and control. Recurrent neovascularization was thought to be the material cause in late recurrent VH. Resident time of anti-VEGF agents in vivo and whether the panretinal photocoagulation had been done fully may be the vital factors of neovascularization. However, fewer studies have testified the half-life of KH902 in vivo, especially serum and ocular level after PPV. With approximately 2.5–4.2 days in rabbit ocular tissues reported by Li et al. (a single intravitreal injection of 0.5 mg/0.05 mL KH902) [25], the half-life of KH902 tended to be shorter than bevacizumab, with a reported vitreous half-life of 4.32–6.61 days in rabbit [26, 27] and 9.82 days in humans' aqueous [28] after a single intravitreal injection of 1.25 mg/0.05 mL bevacizumab at the dose recommended, compared to ranibizumab, with 7.19-day aqueous half-life of 0.5 mg intravitreally injected into human nonvitrectomized eyes [29]. The dosing of angiogenesis inhibitors, lens status, and ocular volume have no obvious impact on drug pharmacokinetics [30, 31]. But with the application of conbercept, panretinal photocoagulation could be done adequately during the surgery so as to decrease late recurrent VH.

With regard to vitreous clear-up time of recurrent VH, postoperative results showed great promise with shorter absorption of early recurrent VH in IVC pretreatment group than in the control group (*P* < 0.001), which may be partly due to more frequent and significantly worse early postoperative VH in control group. In our study, the average vitreous clear-up time of early recurrent VH in preoperative intravitreal conbercept and control group was 5.44 ± 1.94 and 11.31 ± 4.24 days, respectively. There was no significant difference in clear-up time of late recurrent VH (*P* = 0.462).

In the current study, patients that received an IVC injection before PPV did not have better BCVA at 3 months after surgery than patients in control group. However, IVC group showed much better postoperative BCVA within 1 month after surgery and improved BCVA at all the follow-up time. Besides, percentage of good BCVA in IVC group also presented a stable and gradual increase during all the 3-month follow-up time after surgery.

Our study is limited by short follow-up time and lack of other anti-VEGF agents' control. Further multicenter and larger sample studies may be required to assess the efficacy and safety in preventing postoperative VH after diabetic vitrectomy in the longer term.

In the order of importance from primary outcome to secondary outcomes, such as fewer intraoperative and postoperative recurrent VH, shorter absorption of blood, and better VA early after surgery in IVC pretreatment group than in control group without serious adverse events, this randomized prospective study could conclude that that intravitreal injection of conbercept before diabetic retinopathy can reduce the incidence of early postoperative vitreous hemorrhage and can reduce early visual restoration.

## Figures and Tables

**Table 1:** Baseline characteristics of participants with or without conbercept pretreatment.

	Preoperative intravitreal conbercept	Control	*P* value
Number of eyes	54	53	
Sex (male)	27 (62.79%)	24 (53.33%)	0.396
Age (y)	48.63 ± 8.24	49.64 ± 8.71	0.576
Systemic profile			
Type of diabetes			0.517
1	6 (13.95%)	4 (8.89%)	
2	37 (86.05%)	41 (91.11%)	
HbA1c at time of surgery	7.90 ± 1.14	7.63 ± 1.24	0.305
Duration of diabetes (y)	16.67 ± 4.53	15.87 ± 4.77	0.418
Systemic hypertension	11 (25.58%)	11 (24.44%)	1.000
Ocular profile			
Study eye (left/right)	19/35 (35.19%/64.81%)	21/32 (39.62%/60.38%)	0.692
Previous history of laser	11 (20.37%)	16 (30.19%)	0.272
Lens status (pseudophakic/phakic)	11/43 (20.37%/79.63%)	8/45 (15.09%/84.91%)	0.614
Pathogeny			0.869
Nonclearing vitreous hemorrhage	22 (40.74%)	23 (43.40%)	
Diffuse fibrovascular proliferation	27 (50.00%)	24 (45.28%)	
Traction retinal detachment	5 (9.26%)	6 (11.32%)	
Extent of vitreoretinal adhesion grade			0.604
3	17 (31.48%)	19 (35.85%)	
2	19 (35.19%)	21 (39.62%)	
1	18 (33.33%)	13 (24.53%)	
0	0 (0%)	0 (0%)	

HbA1c: glycosylated hemoglobin A1c.

**Table 2:** Surgical procedures and outcomes of participants with or without conbercept pretreatment.

	Preoperative intravitreal conbercept (*n* = 54)	Control (*n* = 53)	*P* value
Severity of intraoperative bleeding grade			0.002
3	3	10	
2	10	22	
1	27	14	
0	14	7	
Recurrent VH			
≤4 weeks	9 (16.67%)	26 (49.05%)	0.000
Severity of recurrent VH grade			0.000
4	0	1	
3	0	2	
2	2	8	
1	7	15	
>4 weeks	6 (11.11%)	8 (15.01%)	0.973
Severity of recurrent VH grade			0.732
4	0	0	
3	0	1	
2	2	3	
1	4	4	

VH: vitreous hemorrhage.
